# Age Related Decline in Cortical Multifocal Flash VEP: Latency Increases Shown to Be Predominately Magnocellular

**DOI:** 10.3389/fnagi.2018.00430

**Published:** 2019-01-18

**Authors:** Alyse Brown, Molly Corner, David Crewther, Sheila Crewther

**Affiliations:** ^1^School of Psychological Science and Public Health, La Trobe University, Melbourne, VIC, Australia; ^2^Centre for Human Psychopharmacology, Swinburne University of Technology, Melbourne, VIC, Australia

**Keywords:** aging (aging), neural efficiency, magnocellular, visual evoked potential (VEP), non-linear dynamics, flicker fusion

## Abstract

As the visual system ages, flicker sensitivity decreases and the latencies of cortical visual evoked potentials (VEP) increase. However, the extent to which these effects reflect age-related changes in the magnocellular (M) and or parvocellular (P) pathways remain unclear. Here, we investigated the relation between flicker fusion frequencies and VEP non-linearities induced by rapid stimulation, as a function of age over 6 decades. The approach, using Wiener kernel analysis of multifocal flash (mf)VEP, allows the extraction of signatures of both M and P processing and hence establishing a neural basis of the known decline in flicker fusion threshold. We predicted that, in a sample of 86 participants, age would be associated with a latency increase in early mfVEP response components and that flicker fusion thresholds, for both low and high contrast stimuli, would relate to the temporal efficiency of the M-generated VEP component amplitudes. As expected, flicker fusion frequency reduced with age, while latencies of early second order peaks of the mfVEP increased with age, but M temporal efficiency (amplitude ratio of first to second order peaks) was not strongly age-related. The steepest increases in latency were associated with the M dominated K2.1 (second order first slice) N70 components recorded at low and high contrast (6.7 and 5.9 ms/decade, respectively). Interestingly, significant age-related latency shifts were not observed in the first order responses. Significant decreases in amplitude were found in multiple first and second order components up to 30 years of age, after which they remained relatively constant. Thus, aging and decline in visual function appears to be most closely related to the response latencies of non-linearities generated by the M pathway.

## Introduction

Changes in visual processing with age have been predominantly associated with temporal processing. Behavioral techniques such as flicker fusion frequency (McFarland et al., 1958; Tyler, 1989; Kim and Mayer, 1994) and visual inspection time (Deary et al., 2010) provide evidence of decrease in rate of visual processing with age and are more evident as cognitive load increases (Owsley, 2011; Ebaid et al., 2017). Such age related impairments have been attributed to changes in the optical aspects of the eye, including the anatomy of the retina and optic nerve conduction rates (Mauk and Buonomano, 2004) and also to occur asynchronously within different cortical areas (Mora et al., 2007; Owsley, 2011). In particular, visually evoked potential (VEP) research shows an increase in the physiological latency of afferent input to primary visual cortex (V1) as a function of age, and as a function of the contrast, spatial frequency and temporal frequency of stimulation (Sokol and Moskowitz, 1981; Celesia et al., 1987; Porciatti et al., 1992; Tobimatsu et al., 1993; Emmerson-Hanover et al., 1994). Simultaneous electrophysiological recordings of retina and V1 have demonstrated increases in the latency response with age to be significantly greater in V1 than in retina (Celesia et al., 1987; Porciatti et al., 1992). In particular the early components (~100 ms) of cortical VEPs show measurable increase in latency with age, although the VEP peak amplitudes remain relatively stable during adulthood. The question of whether any of the major retino-cortical pathways, i.e., magnocellular (M), parvocellular (P) or koniocellular (K), is more affected by aging, has not been reported in human EEG recordings.

An extensive literature exists regarding age effects on the topography of M and P cell types in human retina (Curcio and Allen, 1990; Gao and Hollyfield, 1992) and in the laminae of monkey LGN (Spear et al., 1994) the major thalamic projection site to cortical area V1 (Nassi and Callaway, 2009). In retinal studies Curcio and Allen (1990) initially noted an average 25% reduction in retinal ganglion cell density in older donor eyes within the central 11° of the fovea and hence assumed that aging primarily impacts on P function. Evidence for greater anatomical decline in central P cells is also supported by a comparison of spontaneous discharge rate and optimal temporal frequency in LGN in young and older monkeys (Spear et al., 1994) where only P cells showed age-related changes. On the other hand, recordings from single cells in monkey cortex, showed that the increase in spontaneous discharge rate was present regardless of stimulus properties (Schmolesky et al., 1998; Yang et al., 2008, 2009; Zhang et al., 2008) suggesting that both M and P pathways are altered.

Temporal analysis of the non-linear evoked responses of the retina through Wiener Kernel analysis of multifocal flash electroretinogram (mfERG) was introduced by Sutter (1992) (VERIS, EDI, USA). Wiener kernel analysis takes into account the previous history of stimulation before the current event and hence first and higher order temporal responses to a new stimulus are extracted from the resultant of each patch's previous binary stimulus sequence, as an alternate measure to the more frequently reported pattern reversal analysis (see **Figure 2** for a brief explanation of kernel extraction). A recent clear review of multifocal techniques in ophthalmic electrophysiology (Müller and Meigen, 2016), concludes that there is considerable room for further investigations of non-linear processes. Temporal analysis of the central patch in multifocal flash ERG has shown that second order latencies increase 0.3–0.5 ms/decade over an age range of 18–80 yr, while the first order latencies remain relatively stable (Nabeshima et al., 2002).

In cortical flash mfVEP recordings, it is possible to attribute peaks of the non-linear kernels to the different afferent neural types. Klistorner et al. (1997), using flash mfVEP, demonstrated that the peak amplitudes of the first slice of the second order kernel, K2.1, mimicked the M contrast response function observed in monkey LGN, while the main peak of the second slice of the second order kernel, K2.2 mimicked the monkey's P contrast response function (Derrington et al., 1984; Hubel and Livingstone, 1990). These second order responses also reflect the latency advantage that M pathway has over P in activation of V1 with the major peak of K2.2 showing a longer latency than that of K2.1 by approximately 25 ms in the cortical mfVEP (Klistorner et al., 1997; Sutherland and Crewther, 2010; Jackson et al., 2013).

Recently, a study of a large sample of young adults by Brown et al. (2018) found behavioral support for Klistorner's claim that K2.1 cortical response is dominated by M inputs by demonstrating that higher flicker fusion frequencies correlated with smaller K2.1 component amplitudes. Brown et al. defined a measure of neural efficiency for the M and P pathways, normalizing first and second order responses within each individual, through the ratio measures: M-ratio = K1_N70−P100_:K2.1_N70−P100_ and P-ratio = K1 _N140−P180_:K2.2 _N120−P150_. They found that achromatic flicker fusion frequencies were positively correlated with M-ratios. This definition of efficiency (relatively smaller second order amplitude for higher efficiency) conforms with previous nutraceutical (Bauer et al., 2011; Jackson et al., 2013) claims and individual difference (Bauer et al., 2011; Jackson et al., 2013) studies.

Thus, this study aimed to compare psychophysically measured flicker fusion thresholds and flash mfVEPs recorded from the cortical visual systems of individuals from 18 to 79 years in age, to investigate whether the behavioral decline in temporal processing with age can be linked to changing components of the two major visual pathways. An age-related decline in the latency of early response components and a decrease in temporal efficiency for the M pathway (as measured by the ratio of M generated amplitudes) was predicted.

## Methods

### Participants

Following approval from the Latrobe University Human Research Ethics Committee 86 participants ranging in age from 18 to 79 years were recruited from the university and the surrounding local community. Written informed consent was obtained from all participants in this study. After and exclusion of 10 participants due to low signal to noise ratio, the final sample included 76 individuals, 57 females (age: 41 ± 18 years) and 19 males (age: 45 ± 21 years). A *t*-test revealed that age did not differ between genders (*t*_(74)_ = −0.745, *p* = 0.459). For quantile analyses, participants were evenly split into age groups of young *n* = 26 (18–30 yr: 21 ±.56), middle *n* = 25 (31–55 yr: 42 ± 1.48) and old *n* = 25 (56–80 yr: 65 ± 1.15). All participants indicated on the consent form that they had normal or optically corrected to normal vision, and no history of epilepsy.

### Flicker Frequency Task

The flicker stimulus consisted of 4 achromatic light-emitting diodes (LEDs; A-Bright Industrial Co., China, part AL-513W3c-003 white). Smooth variation in temporal frequency was achieved by the use of VPixx software driving the analog output of a DATAPixx interface device (www.vpixx.com). A gaussian temporal envelope (FWHM = 480 ms) was also employed to smooth the onset and offset of the flicker. A ColorCal II (Camridge Research Systems) colorimeter was used to calibrate and linearise the luminance of each LED with maximum luminance adjusted to 86 cd/m^2^ and a mean luminance of 43 cd/m^2^. Luminance emitted from the 4 LEDs was viewed by the participants via 6 mm diameter optic fiber light guides that were set into a wooden display board in a square diamond array. Light sources were separated by 1° of visual angle when viewed by participants at a distance of 60 cm. Two separate achromatic flicker frequency thresholds were measured using high contrast (75%) and low contrast (5%) temporal modulation. In a 4-way forced choice design, a PEST algorithm (embedded in the VPixx software) was used to estimate flicker threshold at the completion of 32 trials. The high and low contrast flicker tasks were presented in a counterbalenced order to control for practice effects.

This task was completed in a dimly illuminated laboratory environment. During the task, the LEDs remained on continuously. Sounds were used to notify participants of the start and end points of each trial, with a high pitch beep indicating the start of trial, followed by 3 s of target flicker and a low pitch beep to mark the end of the trial. Participants were instructed to use the button box provided to indicate which of the four lights flickered. An initial practice session containing 10 trials that covered flicker frequencies into the threshold ranges was conducted to familiarize participants with the task.

### mfVEP Method

Gold-plated electrodes were placed at the recording site Oz, and at the reference site Fz (10/20 standard positions). A ground electrode was attached to the left earlobe. Electrode preparation was conducted according to ISCEV standards (Odom et al., 2010) and impedances below 2 kΩ were achieved.

**Figure 1:**
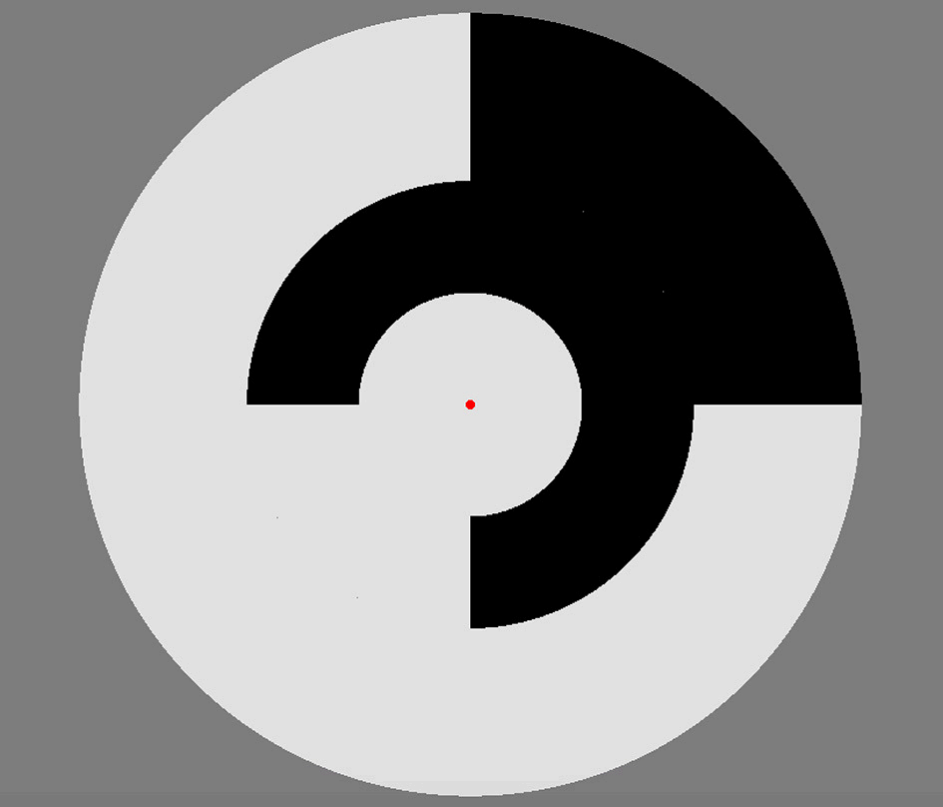
Multifocal stimulus at 96% luminance contrast employed for the VEP recordings. A central circular disk subtended 7° of visual angle was surrounded by two rings (outer diameters 14 and 24.5°) each divided into 4 separate patches. Each patch fluctuated between two luminance levels on the basis of a pseudorandom m-sequence.

Participants were seated at a viewing distance of 70 cm from the stimuli in a dimly lit room. During the recording participants were asked to focus on a central red fixation dot whilst the stimulus was presented. A ViewSonic E90 CRT 21″ monitor with a 75Hz frame-rate was used to display the achromatic multifocal flash stimuli that comprised of 9 luminance-defined patches within a circular display (see Figure 1). The stimulus was created and run in VPixx and employed a DATAPixx interface box for strict video frame registration. Each segment of the stimulus flickered between two luminance levels to a binary pseudorandom m = 14 sequence, with the sequence for each patch maximally shifted resulting in stimuli segments that are mutually decorrelated. The m-sequence allows for an equal occurrence of different binary event patterns during the sequence which results in a similar number of response events for each Wiener kernel analysis (see data pre-processing individual sequence analysis). Participants completed two separate electrophysiological recordings for stimulus contrast presentations 96 and 24%, targeting maximum separability of M and P activation in the second order kernels (Klistorner et al., 1997). These stimuli had mean luminance of 52 cd/m^2^ as measured by a ColorCal (MkII, Cambridge Research Systems) probe. Each contrast presentation condition was broken into 4-time length recording segments of 54.5 s, which was done to prevent fatigue and also allowed participants to rest their eyes. During these breaks participants were asked to blink, look around the room and then close their eyes for the count of 10, participants were then asked to indicate when they felt ready to start the next recording. Visual evoked responses were only recorded from the central stimulus patch that was made large (subtending 7° of visual angle) to improve the signal-to-noise ratio of the macular response and assure that differences in acuity across an aging population was not an issue. Clinical retinotopic research of combined ERG and VEP recording also show disease has the least effect on the central retinal and cortical responses (Hood and Zhang, 2000; Gränse et al., 2004). The surrounding patches act to eliminate the contribution to the visual response from the edges of the central patch.

### Data Pre-processing

**Figure 2:**
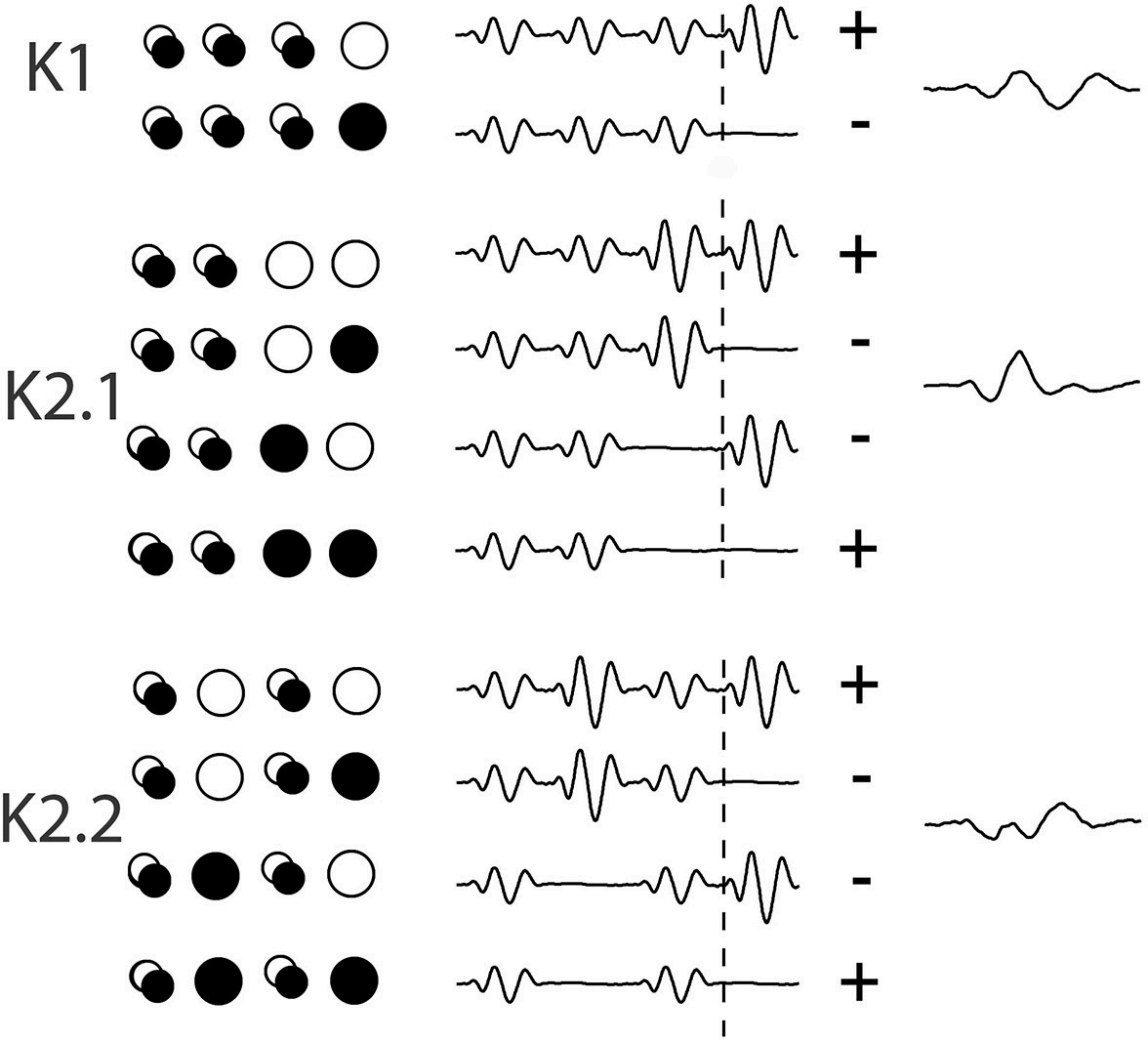
An illustration of the extraction of Wiener kernels using m-sequences via cross-correlations. The circles are a representation of the binary ON/OFF flash stimulation of one stimulus patch across 4 frames of presentation, with a trace of the response wave forms adjacent, artificially spaced for viewing. Over the period of 4 events (53 ms) the singular circles represent the on/off event pattern that contributes to the kernel response. The overlapping circles denote a frame of either polarity. The + and – signs at the end of each set of waves indicates the sign of contribution (addition or subtraction) to the eventual kernel component.

**Figure 3:**
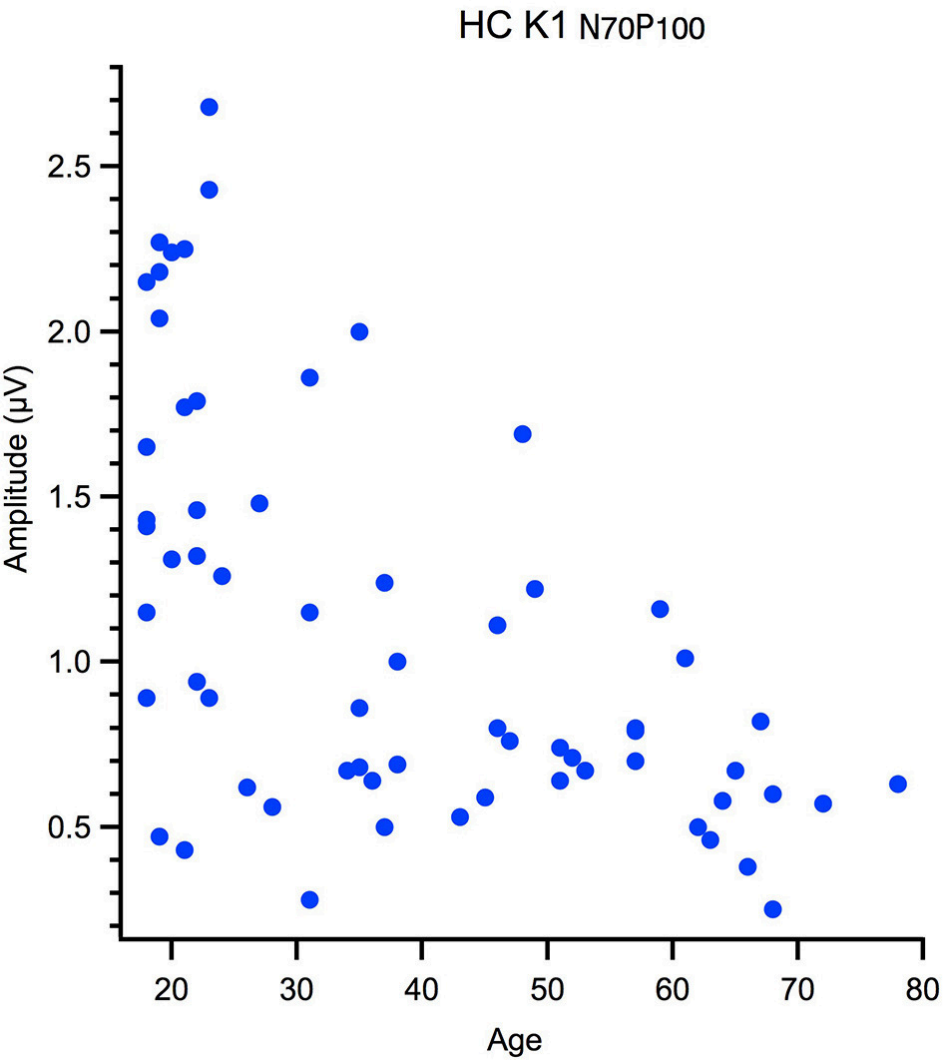
Hetereroscedastic scatter in the relationship between peak to peak amplitude and age of the High Contrast first kernel (HC K1 N70-P100).

Data was collected using Curry-7 (compumedicsneuroscan.com) recording software. The signal was amplified 10,000 times and sampled at 1 kHz and band-pass filtered (1–1 kHz) with a 50 Hz notch filter applied. Over the course of the recording, 16,384 triggers were collected, with triggers being delivered every frame (13.33 ms). For each trigger, a 700 ms epoch was extracted from 200 pre- and 500 ms post-trigger onset. The removal of eye blinks was performed manually in the raw EEG trace and a base line correction using the first 50 data points was applied. The triggers correspond to different combinations of on/off patterns within the central patch, and these allow for the extraction of the first and second order VEP Wiener kernels K1, K2.1, and K2.2 (for more on the Wiener kernel expansion used here, see **Figure 3** and the article by Sutter (2000). Briefly, in the binary White/Black m-sequence, the first order response (K1) corresponds to the average of all responses to a white stimulus (R_W_) minus the average of all responses to a black stimulus (R_B_), i.e., 0.5^*^(R_W_-R_B_). The second-order first and second slice responses (K2.1, K2.2) are the temporal non-linearities of the visual response that takes into account the history of stimulation. Response K2.1 represents analysis across two consecutive frames, this time all responses when a transition has occurred (R_BW_ + R_WB_) are averaged minus the average of responses when no transition occurs (R_BB_ + R_WW_), i.e., 0.25^*^(R_BB_ + R_WW_-R_BW_ + R_WB_), while K2.2 has the same comparison with an additional intervening frame of either polarity (i.e., the comparison is one frame further back–see Figure 2).

Using IGOR Pro (Wavemetrics, USA) amplitudes and latencies of peaks were extracted for participants' first and second order VEP responses. Mean average waves of the 76 participants were calculated for each kernel. Bonferroni corrections for the multiple comparisons were employed and Mahalanobis distances were used to detect outliers. To reduce between-subject variation in recording conditions such as skull thickness and muscle artifact, ratios of the first order to second order amplitudes of the prominent M and P generated peaks were calculated for each participant. The M ratio has been defined as K1_N70−P100_:K2.1_N70−P100_ and the P ratio has been defined as K1 _N140−P180_:K2.2 _N120−P150_, thus the larger the ratio the higher the neural efficiency demonstrated (Brown et al., 2018).

### Data Analysis

Correlational analyses with age were run for peak latency data and M and P ratios. These data were not normally distributed, hence one-tailed Spearman's tests were used, with alpha was corrected for multiple comparisons. Outliers were removed from the correlation based on Mahalanobis D squared distance of 13 and above (Rasmussen, 1988). The amplitude data, measured from peak-to-peak, was not suitable for a linear analysis as the data had a heteroscedastic spread for an example see Figure 3. Thus, a between groups analysis of participants aged 18–30 yr (young) 31–55 yr (middle) and 56–80 yr (old) using Welch's test was conducted. The significance level was corrected for multiple comparisons (12) to α = 0. 004.

## Results

### Age and VEP

**Figure 4:**
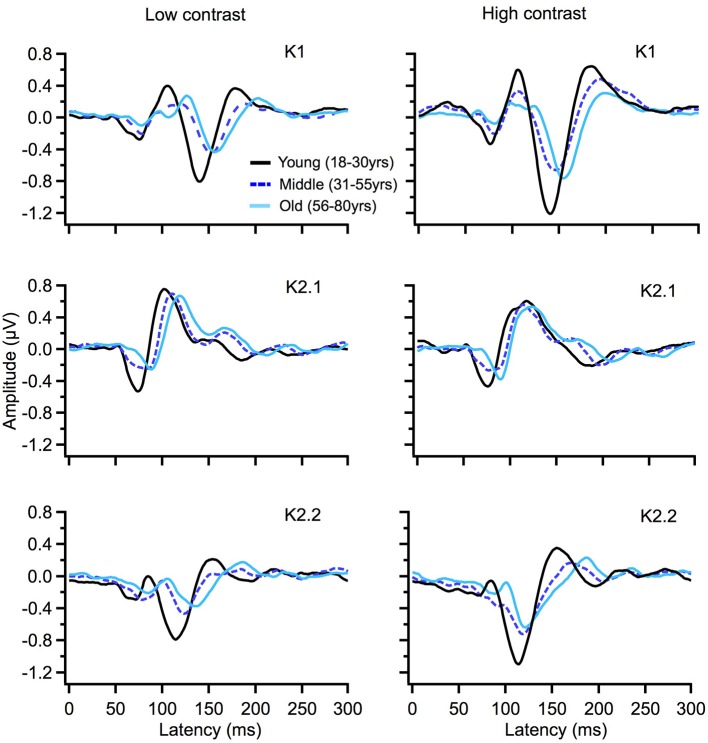
VEP group average responses for young (black), middle (dark blue, dashed) and old (light blue) groups. The left column shows responses for low contrast (24%) while the right column shows responses for high contrast (96%) stimulation.

To illustrate changes in the VEP waveforms with age, the mean average kernels for the 18–30 yr (young) 31–55 yr (middle), and 56–80 yr (old) age groups are displayed in Figure 4 and the corresponding mean and standard error group values are displayed in Table 1. In the second order kernel components, there is an observable trend of latency increasing with age (see Figure 4, Table 1) including several age-related changes in latency that are significant (see Table 2).

**Table 1:** Peak VEP latency (ms) by age group.

	**Low contrast (M** **±** **SE)**			**High contrast (M** **±** **SE)**		
	**Young**	**Middle**	**Old**	**Young**	**Middle**	**Old**
**K1**						
N70	74.4 ± 2.0	80.3 ± 1.9	78.1 ± 1.3	79.2 ± 1.9	79.2 ± 1.6	82.8 ± 3.1
P100	105.4 ± 2.4	106.5 ± 2.9	109.9 ± 3.9	106.7 ± 1.9	105.3 ± 2.0	110.2 ± 3.1
N140	140.5 ± 1.7	137.0 ± 4.0	139.8 ± 5.1	140.8 ± 1.9	140.4 ± 2.6	141.5 ± 4.6
P175	180.1 ± 3.6	174.1 ± 6.5	177.9 ± 7.9	186.1 ± 2.5	184.1 ± 4.1	182.2 ± 7.2
**K2.1**						
N70	73.7 ± 1.3	81.0 ± 1.9	90.8 ± 1.5	74.1 ± 1.5	81.3 ± 1.9	90.0 ± 1.8
P100	104.3 ± 2.2	110.7 ± 2.2	115.1 ± 2.1	104.3 ± 2.5	112.7 ± 2.7	116.4 ± 2.4
N135	130.0 ± 2.9	133.1 ± 3.8	141.4 ± 3.6	132.0 ± 4.6	141.5 ± 5.3	149.8 ± 5.7
**K2.2**						
N70	69.4 ± 1.7	76.7 ± 2.1	80.0 ± 2.8	68.6 ± 2.3	77.4 ± 1.9	83.6 ± 2.2
P85	90.7 ± 2.1	96.1 ± 2.4	103.1 ± 2.2	90.0 ± 1.7	90.3 ± 2.3	100.1 ± 2.4
N110	117.2 ± 2.1	120.6 ± 2.5	125.6 ± 2.9	117.0 ± 1.8	114.0 ± 1.9	117.3 ± 1.1
P150	149.9 ± 1.8	152.5 ± 3.9	171.4 ± 4.2	155.0 ± 2.1	158.1 ± 3.4	165.1 ± 4.1

**Table 2:** Correlations between VEP peak latency components and age.

**Latency component**	**Low contrast**			**High contrast**		
**K1**						
N70	*r =* 0.218	df(64)	*p =* 0.040	*r =* 0.046	df(65)	*p =* 0.357
P100	*r =* 0.128	df(64)	*p =* 0.155	*r =* 0.062	df(65)	*p =* 0.311
N140	*r =* 0.147	df(64)	*p =* 0.121	*r =* 0.133	df(65)	*p =* 0.143
P175	*r =* 0.063	df(64)	*p =* 0.310	*r =* 0.093	df(65)	*p =* 0.228
**K2.1**						
N70	*r =* 0.674^**^	df(62)	*p <* 0.000	*r =* 0.588^**^	df(65)	*p <* 0.000
P100	*r =* 0.315	df(64)	*p <* 0.005	*r =* 0.346^**^	df(64)	*p <* 0.002
N135	*r =* 0.187	df(64)	*p =* 0.068	*r =* 0.204	df(65)	*p =* 0.050
**K2.2**						
N70	*r =* 0.401^**^	df(63)	*p <* 0.000	*r =* 0.507^**^	df(64)	*p <* 0.000
P80	*r =* 0.421^**^	df(63)	*p <* 0.000	*r =* 0.401^**^	df(65)	*p <* 0.000
N110	*r =* 0.274	df(62)	*p =* 0.015	*r =* 0.235	df(63)	*p =* 0.031
P150	*r =* 0.393^**^	df(63)	*p <* 0.001	*r =* 0.320	df(63)	*p =* 0.005

One tailed Spearman's correlation,

** *indicates statistical significant at p < 0.002 with alpha corrected for multiple comparisons*.

### Analysis of Latency and Age

The correlational analysis showed that age correlated positively with the latencies of the first two peaks of the K2.1 and K2.2 waveforms, for both the low and high contrast conditions (Table 2). There was one further significant correlation found between age and latency in the K2.2 P150 component for the low contrast condition. The strength of the correlations was greater for the first negative peak (N70), compared to the following positive peak (P110), with the exception of the K2.2 response at low contrast.

**Figure 5:**
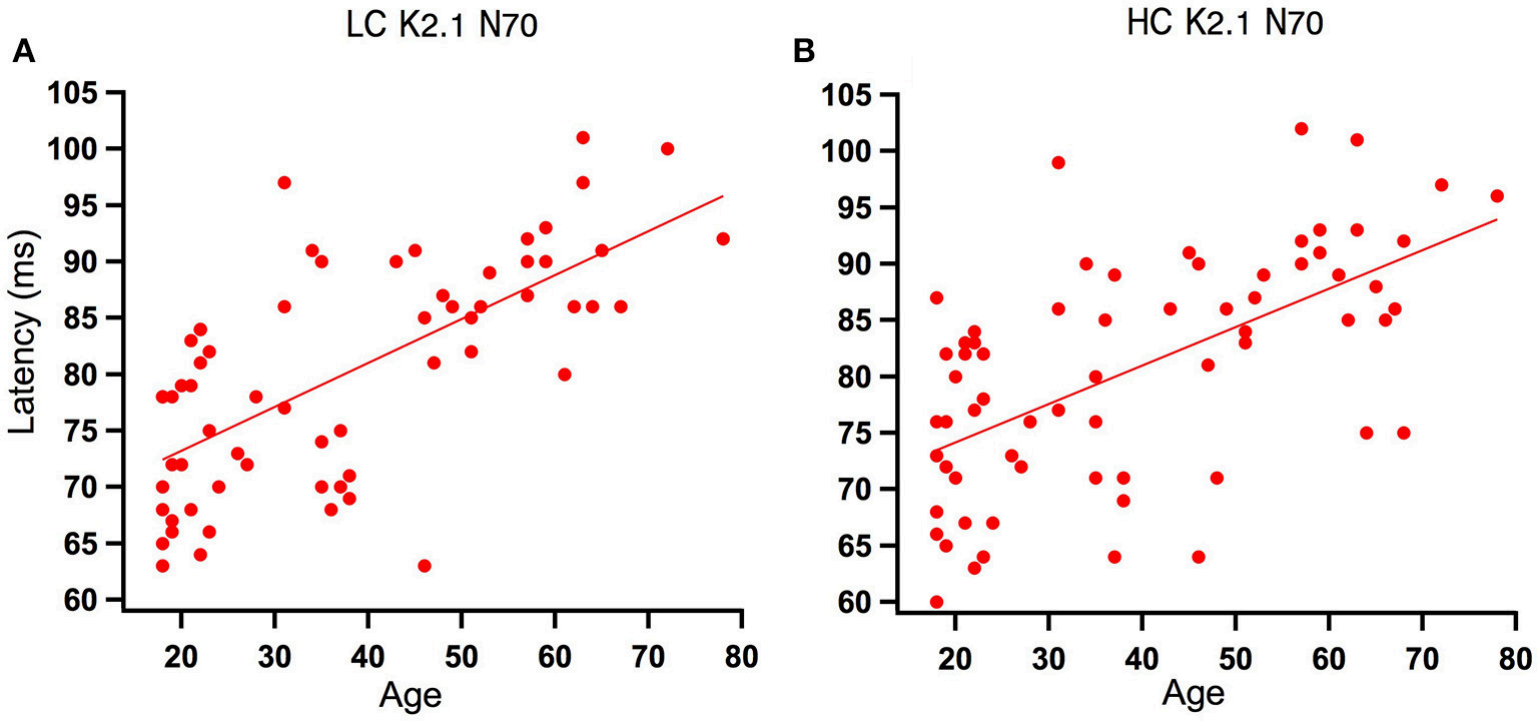
Scatter plot of latency versus age for the Magnocellular nonlinearity K2.1_N70_ at Low and High Contrast. **(A)** At low contrast (LC), one tailed Spearman's correlation showed that the K2.1_N70_ latency component was significantly correlated with age (*r* = 0.674, *p* < 0.000) and explains 46% of the variance. **(B)** At high contrast (HC), one tailed Spearman's correlation showed that the K2.1_N70_ latency component was significantly correlated with age (*r* = 0.588, *p* < 0.000) and explains 36% of the variance.

Analysis of the regression slope showed that the two correlations with the fastest rate of latency increase with age were the K2.1 N70 components for the high and low contrast stimulus conditions. In the low contrast condition, response latency increased 6.7 ms per decade and in the high contrast condition response latency increased 5.9 ms per decade (see Figure 5). No significant age-related latency changes were observed in the first order kernels, with the mean latency increase for the K1_N70_ peak being 2.2 ms/decade.

### Analysis of Amplitude and Age

**Figure 6:**
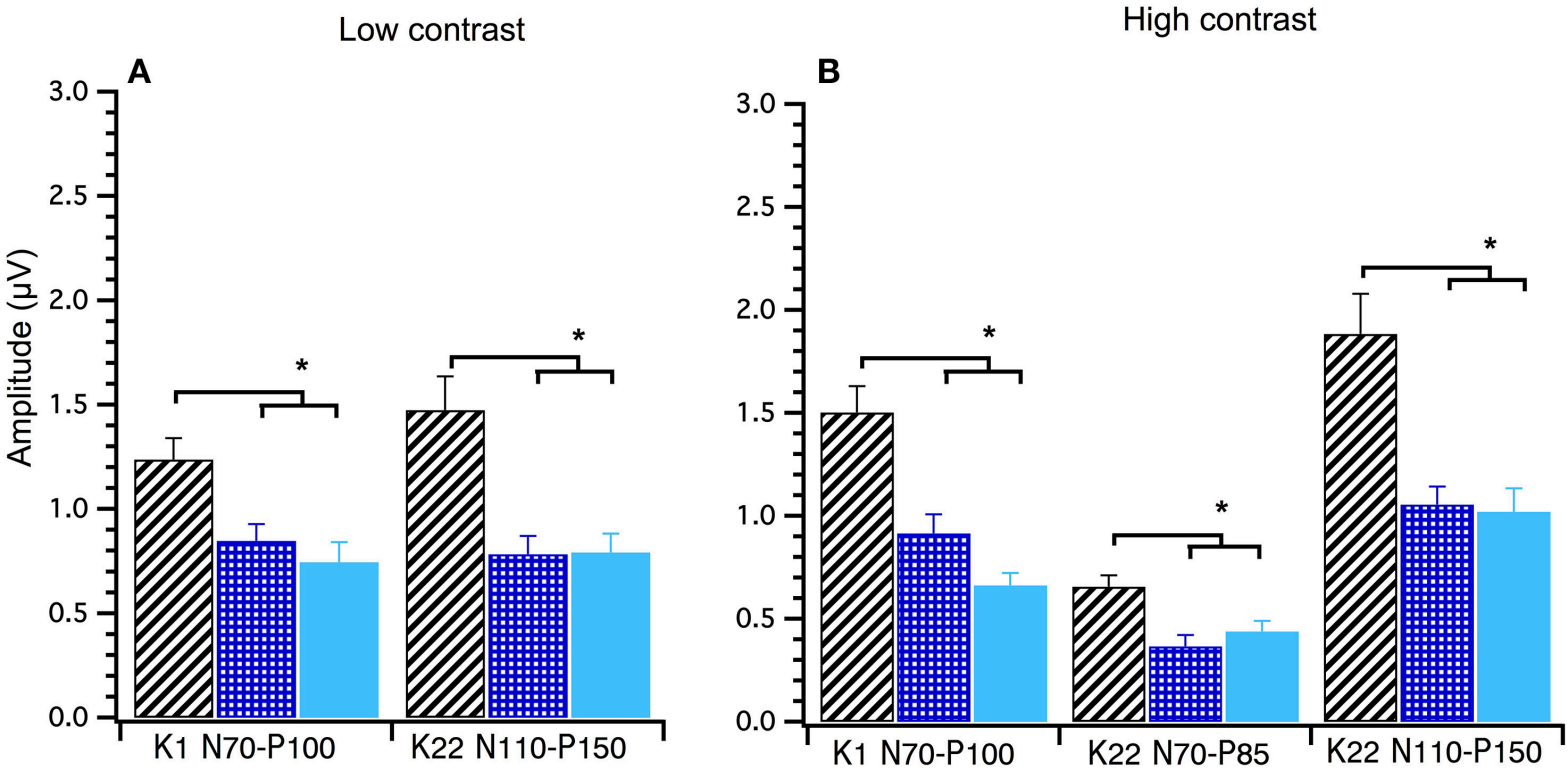
Group amplitude means and standard deviations comparing young (diagonal lines), middle (checked) and old (solid) participants at Low and High Contrast. Between groups ANOVAs were conducted. Both the Low **(A)** and High **(B)** contrast graphs show that the young group's amplitudes are significantly higher (*p* < 0.003) than the middle and old groups in these *post hoc* tests. ^*^Indicates statistical significant at *p* < 0.004.

A between groups (young, middle and old) analysis of peak-to-peak amplitude data was conducted using Welch's test. Significant main effects of group were found for the following amplitudes at high contrast; K1_N70−P100_ *F*_(2, 40)_ = 18.00, *p* < 0.001, K22_N70−P85_ *F*_(2, 40)_ = 7.34, *p* < 0.000 and K22_N110−P150_ *F*_(2, 39)_ = 8.14, *p* < 0.001 and at low contrast; K1 _N70−P100_ *F*_(2, 39)_ = 6.71, *p* < 0.003 and K22_N110−P150_ *F*_(2, 39)_ = 7.75, *p* < 0.001. Games–Howell *post-hoc* testing showed that the young group had significantly larger amplitudes than the mid and oldest groups. These data show that amplitudes decrease until around 30 years after which time amplitudes stabilize with age (see Figure 6).

**Figure 7:**
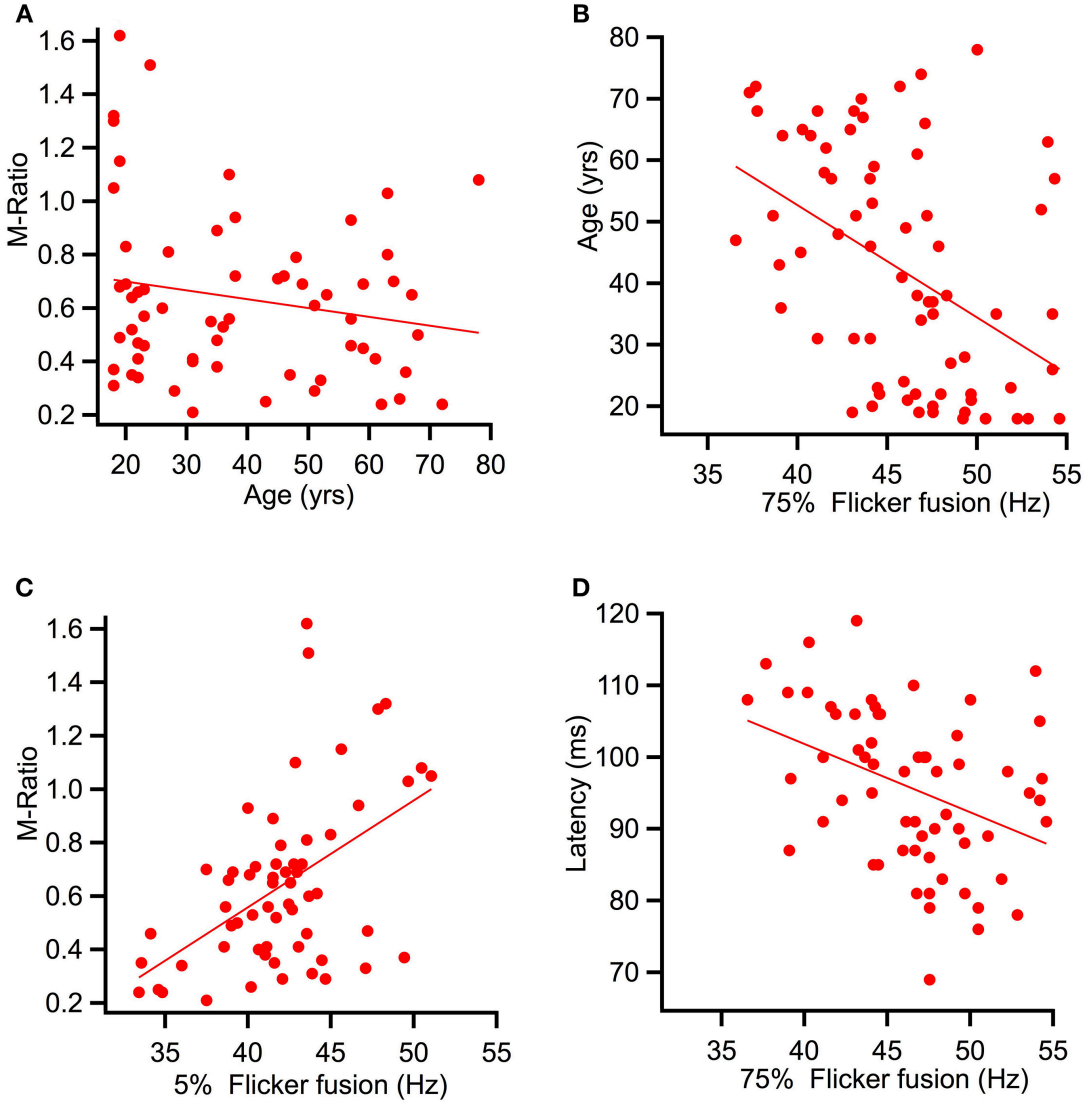
Scatter plots showing relationships between variables predicted to correlate. **(A)** shows a relationship between M-ratio and age to be non-significant (*r* = −0.186, *p* = 0.071). **(B)** Age significantly correlated with flicker fusion (*r* = 0.362, *p* < 0.002). **(C)** and **(D)** present the multiple regression results of the variables found to account for largest amount of variance in flicker fusion threshold. **(C)** M-ratio accounted for the most variance in low contrast flicker fusion at 24.8%(*r* = 0.498, *p* < 0.001) and **(D)** low contrast K2.2_P80_ peak latency accounted for the most variance in the high contrast flicker fusion at 14.9% (*r* = −0.433, *p* < 0.000).

Spearman's correlational analysis of age and ratios of M and P activation showed that age did not significantly correlate with the P ratio (*r* = −0.055, *p* = 0.332) nor with the M-ratio (*r* = −0.186, *p* = 0.071). Figure 7A shows a scatter plot of age against M-ratio.

### Variance in Flicker Fusion Threshold

Spearman's correlational analysis between age and flicker fusion frequency revealed significant negative correlations with age at high contrast (*r* = −0.438, *p* < 0.001 m, 19% of variance) and at low contrast (*r* = −0.304, *p* < 0.005, 9% of variance). Spearman's correlational analysis between flicker fusion and M ratio showed a significant positive correlation in both low (*r* = 0.498, *p* < 0.001) and high (*r* = 0.362, *p* < 0.002) contrast flicker. Additional correlations were run with P ratio and flicker fusion threshold however, no significant results were found. For displays of the strongest reported relationships between flicker fusion and M-ratio and flicker fusion and age see Figures 7B,C.

An exploratory analysis between flicker threshold and all latency components was conducted after correcting alpha (0.006) for multiple comparison (*n* = 8). Amplitude was not included in the analysis as these data are not linear. High contrast flicker fusion correlated with components K2.1_N70_ (*r* = −0.339, *p* < 0.004), K2.2_N70_ (*r* = −0.325, *p* < 0.005), and K2.2_P80_ (*r* = −0.433, *p* < 0.000) recorded at low contrast and with K2.2_N70_ (*r* = −0.338, *p* < 0.004) and K2.2_P80_ (*r* = −0.325, *p* < 0.005) recorded at high contrast. Low contrast flicker fusion threshold correlated with components K2.1_P100_ (*r* = −0.300, *p* < 0.005), K2.2_N70_ (*r* = −0.393, *p* < 0.001), and K2.2_P80_ (*r* = −0.425, *p* < 0.000) recorded at low contrast while no significant correlations were found with the high contrast recording.

To assess what variables were contributing most to the variance in flicker threshold, a multiple linear regression analyses was run. Age, M-ratio, low contrast K2.1_N70_ latency, and low contrast K2.2_P80_ latency were added to the regression analysis of high contrast flicker fusion. All variables were entered at once in a stepwise regression where results showed that for high contrast flicker fusion, the regression model explained a significant amount of variance in threshold at stage one with K2.2_P80_ latency entered (*F*_(1, 57)_ = 9.99, *p* < 0.003, *R*^2^ = 0.149) and at stage two where M-ratio was entered (*F*_(1, 56)_ = 5.76, *p* < 0.020, *R*^2^ = 0.229) while K2.1_N70_ latency and age did not significantly contribute to the model and were removed. The R square change showed that the K2.2_P80_ latency explained 14.9% of variance over and above the intercept, and M-ratio contributed an additional 7.9% to the variance over and above stage 1.

To assess the variance contributing to low contrast flicker fusion, the variables age, M-ratio, low contrast K2.1_P100_ latency, and low contrast K2.2_P80_ latency were added into a second regression analysis. Again, the choice of latency component was based on the strongest correlating K2.1 and K2.2 latency component with low contrast flicker fusion. All variables were entered at once in a stepwise regression. Results showed that for low contrast flicker fusion, the regression model explained a significant amount of variance in threshold at stage one with M-ratio entered (*F*_(1, 58)_ = 19.16, *p* < 0.001, *R*^2^ = 0.248) and at stage two where K2.2_P80_ latency was entered (*F*_(1, 57)_ = 7.05, *p* < 0.010, *R*^2^ = 0.331) while K2.1_P100_ latency and age did not significantly contribute to the model and were removed. The R square change showed that the M-Ratio explained 24.8% of variance in low contrast flicker over and above the intercept, and K2.2_P80_ latency contributed an additional 8.3% to the variance over and above stage 1. For displays of the strongest contributing variable to high and low contrast flicker fusion see Figures 7C,D.

## Discussion

Non-linear flash mfVEPs and behavioral measures of flicker fusion have been used to explore changes in M and P generated temporal function as well as the different kernel responses with age. While previous aging studies of M and P properties have largely been anatomical and restricted to retina and LGN (Curcio and Allen, 1990; Gao and Hollyfield, 1992; Spear et al., 1994), the current study has combined psychophysical function through flicker fusion together with the non-linear mfVEP recordings from visual cortex. The results of this study support predictions that the M dominated responses recorded from cortex are more age affected than the P dominated responses. While flicker fusion correlated with the M-ratio (K1_N70−P100_:K2.1_N70−P100_) as seen in Brown et al. (2018), this relationship was not additionally affected by age which was contrary to our hypothesis.

As expected results from our sample aged 18 to 79 years, are consistent with evidence that the latency of early cortical responses increase with age (Sokol and Moskowitz, 1981; Celesia et al., 1987; Tobimatsu et al., 1993; Emmerson-Hanover et al., 1994). In respect to amplitude, there was a significant difference between the young group (18–30) compared to the middle and old groups. This difference was driven by a subgroup of young participants producing very high amplitudes. Indeed, age related amplitude changes are not reported in past cortical evoked response research examining age changes (Sokol and Moskowitz, 1981; Celesia et al., 1987; Porciatti et al., 1992; Tobimatsu et al., 1993; Emmerson-Hanover et al., 1994). The predicted relationship between the M-ratio which is a measure of scaled first to second order amplitudes and age was also not found.

The most compelling age-related changes presented here were the large increases in the second order latency peaks. While the observed latency shifts with age in the early peak latency window of <100 ms is in line with the relevant VEP research, the extent of the latency shift reported in this study is dramatically greater that the total 1–3 ms latency increase reported from age 18 to 80 years previously found (Sokol and Moskowitz, 1981; Celesia et al., 1987; Tobimatsu et al., 1993; Emmerson-Hanover et al., 1994). This study shows that early second order kernel peaks of K2.1_N70_ recorded at high and low contrast were found to have the steepest latency increases with age, 6.7 and 5.9 ms/decade, respectively. Early N70 and P80 latency peaks of the second order kernel K2.2–identified as magnocellular in origin (Klistorner et al., 1997; Sutherland and Crewther, 2010; Jackson et al., 2013) were also found to increase significantly with age for both low and high contrast conditions. The source of the longer latency K2.2_P150_ peak that also showed latency increases with age is unknown. However, given that such a correlation was not observed at high contrast (where P contributions would be expected to contribute to a greater extent), and recognizing that the duration of the event related cortical disturbance lasts from about 50 ms to at least 300 ms, suggests that the K2.2_P150_ may just be the continuation of the M contribution to the K2.2 rather than an age-related P component. Thus, on the whole, M dominated non-linear responses are evidently more pronounced than the P generated responses.

A further observation in this study was that latency increases with age were only found in the second order responses and not in the first order peaks that showed relatively stable latencies with age. This was also found to be the case in multifocal flash ERG aging research that showed over a comparative age range only second order latencies increasing 0.3 to 0.5 ms each decade in a sample established as having normal ocular function (Nabeshima et al., 2002). Thus, only a small percentage of the age driven latency increases detected at cortex in this study are due to retinal changes. While clinical visual screenings were not conducted in this study, we would expect that latency increases due to optical or retinal age related changes reported in previous studies (Sokol and Moskowitz, 1981; Celesia et al., 1987; Porciatti et al., 1992; Burton et al., 1993) would have had a general latency effect observable in all kernels including K1.

It is currently unresolved why mfVEP K1 latencies remain stable with age while second order latencies increase relatively rapidly with age (Nabeshima et al., 2002). The nature of second order mf flash VEP responses reflects the effects of prior frames of stimulation with the possibility of neural feedback affecting waveforms. Furthermore, second order kernels possibly reflect an accumulation of age-affected processes, including age-related myelination changes (Peters, 2002), age-related attentional delay (Kutas et al., 1994) and age-related degree of gray matter cortical involvement in signal generation (Price et al., 2017). However, to date there is little theoretical understanding of the relation between latencies of the first and higher order non-linear components.

Similar dramatic increases in latency has been reported in cells recorded from cortex in a sample of old compared to young monkeys (Wang et al., 2004). Wang found age did not affect all responses, with latency increases tied more to the V1 supragranular layer and V2 whereas 4C layers (inputs for M and P geniculate afferents) of V1 demonstrated stable latencies. These differing response origins shown in Wang's research suggest a possible explanation for why only second order latencies might show different age-related changes compared with first order. The first order kernel K1 is the linearized approximation to the response. We suggest that the higher order kernels, allowing contributions from other neural populations, as feedback, would likely show more non-linear power than the response if it were limited to just the input layers of cortical area V1.

Finally, in exploring the relationship between the three measures of this study age, mfVEP, and flicker fusion, these data provided mixed support for the initial predictions. Age did correlate with a decline in flicker fusion thresholds, however it was not among the strongest predictors of flicker fusion. Interestingly, this study found that the greatest predictor of low contrast flicker variance was the M-ratio, replicating the correlation found in Brown et al. (2018). For high contrast flicker the M-driven K2.2_P80_ latency component recorded at low contrast, predicted the greatest percentage of the variance. Contrary to prediction, no significant correlation was found between age and M-ratio suggesting that the relationship between M-ratio and flicker threshold is independent from that between flicker threshold and age. The concluding analysis found that the M-dominated K2.2_P80_ latency was the closest cortical response component to predicting shared variance of both flicker threshold and age.

In conclusion, the findings of this study and its consideration of non-linear temporal analysis of multifocal cortically evoked responses, add a new level of understanding to age related behavioral changes. The study has demonstrated that age impacts more on mfVEP latencies originating from the M subcortical pathway than from the P subcortical pathway. Lastly, this research also brings attention to the mechanisms behind the latencies of higher order kernels and how aging might alter them.

## Author Contributions

AB was the primary contributor to this study and was involved in the design, research theory, data collection, and write up. MC was an honors student who helped with the data collection. DC and SC co-supervised this study and were involved in the design and the development of the theory and helped with the write up of the manuscript.

### Conflict of Interest Statement

The authors declare that the research was conducted in the absence of any commercial or financial relationships that could be construed as a potential conflict of interest.
